# Failure rates and clinical outcomes of synthetic meniscal implants following partial meniscectomy: a systematic review

**DOI:** 10.1186/s43019-022-00155-1

**Published:** 2022-06-13

**Authors:** Suraj Kohli, Jonas Schwenck, Ian Barlow

**Affiliations:** Department of Trauma and Orthopaedics, Dorset County Hospital NHS Foundation Trust, Williams Avenue, Dorchester, DT12JY Dorset UK

**Keywords:** Knee, Meniscus, Arthroplasty, Osteoarthritis, Implant, Scaffold

## Abstract

**Background:**

Meniscal injury is one of the most common indications for knee surgery. The advent of meniscal repair techniques has facilitated meniscal preservation in suitable cases. Meniscal substitution with scaffolds may be advantageous following partial meniscal resection. There are three main scaffolds in current clinical use; Collagen Meniscal Implant (CMI Stryker Corporation, Kalamazoo, MI, USA), Actifit (Actifit, Orteq Ltd, London, UK) and NUsurface (Active Implants, LLC). The purpose of this systematic review was to compare clinical outcomes and failure rates of patients who have had implantation with these meniscal scaffolds.

**Methods:**

MEDLINE and EMBASE databases were searched for studies that included patients who had surgical implantation with Actifit or CMI. Eligibility criteria included papers that described both clinical outcomes and failure rates of these implants, a mean follow up of 5 years and studies published in English. A Google search was also performed to identify any grey literature.

**Results:**

Five Level IV studies were found for Actifit. One Level II, one Level III and four Level IV studies were found for the CMI implant. One Level II study was identified for the NUsurface scaffold with a follow-up 12 months and was included for completeness. Overall, 262 patients were treated with Actifit, 109 with CMI and 65 with NUsurface. Failure rates for Actifit were 18% (range 6.3–31.8%) with a mean follow up of 66.8 months, and for CMI 6.5% (range 0–11.8%) with a mean follow up of 97.1 months. The NUsurface failure rate was 16.9% at 12 months. Clinical outcomes such as VAS, Tegner and Lysholm scores improved significantly post-operatively. However, there was a high volume of concurrent procedures, such as anterior cruciate ligament reconstructions and high tibial osteotomies in each study group; 118 (45%) for Actifit and 53 (45%) for CMI.

**Conclusion:**

The evidence for meniscal scaffold use is insufficient to suggest that they could potentially improve clinical outcomes in patients post-meniscal resection. This is largely due to the high proportion of concurrent procedures performed at index procedure for both CMI and Actifit. On the basis of current evidence, the use of meniscal scaffolds as a sole treatment for partial meniscal defects cannot be recommended, owing to the relatively high failure rate and paucity of clinical data.

## Introduction

Meniscal injury is one of the most common indications for knee surgery [1]. The advent of meniscal repair techniques has facilitated meniscal preservation in suitable cases [2]. The extent of meniscal resection, however, is proportional to the risk of developing secondary osteoarthritis [3]. Meniscal preservation techniques have been developed to reduce this risk. The success of meniscal repair depends on location and blood supply, with peripheral or ‘red on red’ or ‘red on white’ tears being amenable to surgery [4]. The majority of meniscal tears are, unfortunately, central ‘white on white’ tears with poor blood supply. In these cases, partial meniscectomy is necessary. Meniscal allograft transplantation (MAT) can be implanted following complete meniscal loss, and recently meniscal scaffolds have been developed to replace partial defects [5, 6].

**Fig. 1:**
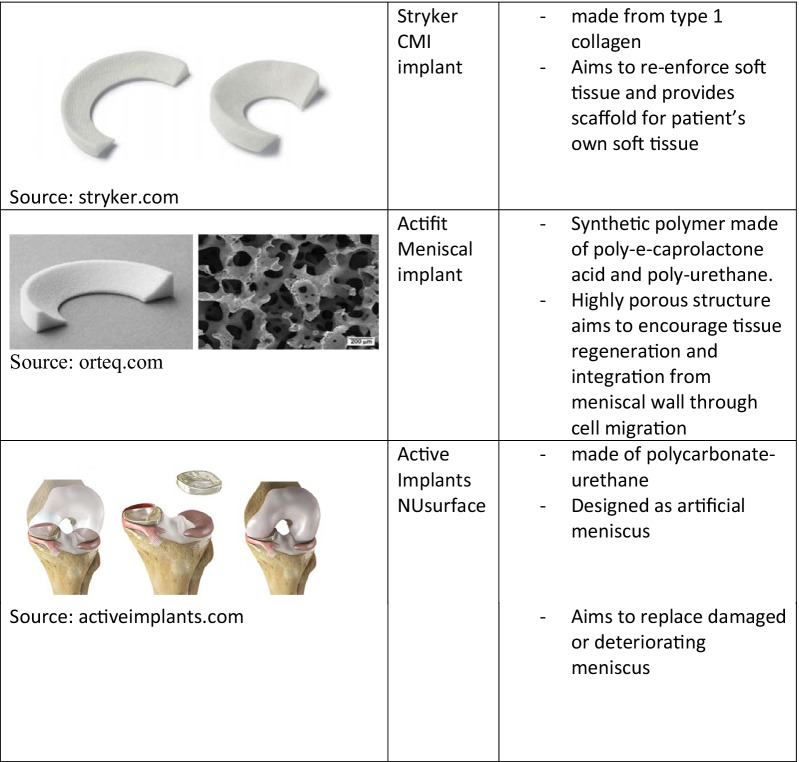
Summary and photographs of meniscal implants

Meniscal scaffolds provide a template for cells and may allow the formation of meniscal-like tissues [7]. There are three main implants in current clinical use. Collagen Meniscal Implant (CMI Stryker Corporation, Kalamazoo, MI, USA) is a collagen scaffold harvested from bovine Achilles tendons, which allows the ingrowth of cells into the menisci [8, 9]. It requires an outer rim of meniscus with an attachment to the anterior/posterior horn [10]. The Actifit (Actifit, Orteq Ltd, London, UK) implant is a synthetic scaffold composed of polyurethrane (20%) and prolactone (80%), [7] which is biodegradable, with a predicted degradation time of 4–6 years [5]. More recently, a polymeric medial implant, the NUsurface (Active Implants, LLC) has been developed [11]. NUsurface is a polyurethrane product made from ultra-high-molecular-weight polyethylene [11] (see Fig. 1).

The goals of meniscal surgery are to improve pain and stability, restore activity levels and reduce the risk of secondary osteoarthritis [12]. The role of meniscal scaffolds in attaining these goals is still uncertain. The purpose of this systematic review is to compare the current evidence of clinical outcome and failure rates of patients who have had surgical implantation of the Actifit, CMI, or NUsurface scaffold.

## Methods

A literature search was performed using the OVID web interface. MEDLINE and EMBASE databases were searched for studies related to outcomes following implantation with the Actifit/polyurethrane implant up to 15 November 2020. The review was performed in accordance with PRISMA guidelines [13]. Terms searched for included ‘meniscus’, ‘synthetic’ and ‘implant. A Google search was also performed to identify any grey literature. The initial search yielded 142 studies. Subsequently, titles and abstracts were reviewed according to the inclusion and exclusion criteria.

A separate literature search was also performed to identify papers related to the CMI implant. Search terms such as ‘meniscal scaffold’, ‘collagen meniscal implant’ and ‘CMI’ were used. This search yielded 532 studies. Titles and abstracts were reviewed for both categories and included/excluded on the basis of the following criteria outlined below.

One reviewer (S.K.) conducted the original literature research as well as the inclusion and exclusion of articles on the basis of the criteria. Subsequently, the included articles were assessed by two reviewers (S.K. and J.S.) and the results were collated. Studies were excluded on the basis of the following criteria: (1) studies not written in English, (2) only abstract or conference data, (3) animal studies.

Individual patient inclusion criteria varied within the papers selected, and indication for meniscal repair included both traumatic and degenerative causes.

All papers included for this review addressed the use of partial meniscal implants; however, depending on the implant used, these could be lateral or medial.

## Outcomes

Studies were included if they reported pre- and post-operative clinical outcomes of patients post-implantation and if they reported failure/reoperation rates. Each study was then assessed and reviewed by two reviewers. Twelve studies were included: five Actifit and six CMI studies (both with a mean follow-up of 5 years), and one NUsurface with a follow-up of 1 year.

## Results

Five Level IV studies were found for Actifit. One Level II, one Level III and four Level IV studies were found for the CMI implant. One Level II study was identified for the NUsurface scaffold. The studies with patient demographics are summarised in Table 1.

**Table 1:** Summary of patient demographics, follow-up and lesion location

	Paper	Level of evidence	Patients	M/F	Location of lesion, medial/lateral	Mean follow-up (months)	Mean patient age
Actifit	Leroy et al. [14]	IV	15	7/8	6/9	72	30
	Dhollander et al. [15]	IV	44	24/20	29/15	60*	32.13
	Toanen et al. [16]	IV	155	109/46	101/54	60*	33.7
	Monllau et al. [17]	IV	32	25/7	21/11	70.2	41.3
	Filardo et al. [18]	IV	16	9/7	12/4	72	45
Total			**262**	**174/88**	**169/93**		
Means						**66.8**	**36**
CMI	Zaffagnini et al. [19]	II	17	17/0	17/0	135	38
	Steadman et al. [20]	IV	8	8/0	8/0	69.6	40
	Zaffagnini et al. [21]	IV	8	8/0	8/0	81.6	31
	Bulgheroni et al. [22]	III	17	13/4	17/0	116.4	32.9
	Bulgheroni et al. [23]	IV	34	25/9	34/0	60*	39
	Monllau et al. [24]	IV	25	20/5	25/0	120*	29.2
Total			**109**	**91/18**	**109/0**		
Means						**97.1**	**35**
NUsurface	McKeon et al. [11]	II	**65**	**41/24**	**65/0**	**12**	**48.7**

*Minimum follow-up rather than mean

Bold represents mean and total values for the individual subgroups of data

All of the studies listed had a greater propensity of male to female subjects. All of the CMI and NUsurface implant lesions were medial, whereas the Actifit implant was used for medial (169) and lateral lesions (93). The follow-up of patients ranged from 60 to 72 months for Actifit, 60–120 months for CMI, and up to 12 months for the NUsurface scaffold. Overall, there were more patients treated by Actifit (262) compared with the CMI (109) and NUsurface (65) implant. Mean follow-up times were 66.8 months for Actifit, 97.1 months for CMI, and 12 months for NUsurface. Mean age was 36 years for Actifit, 35 years for CMI and higher for NUsurface at 48.7 years.

### Patient inclusion and exclusion criteria

The inclusion and exclusion criteria for these studies are summarised in Table 2. Two of the included CMI studies did not state exclusion criteria [21, 22]. Although there is a significant variability in criteria, the majority of studies included patients with irreparable meniscal tears (acute or chronic were both included), and excluded patients who had evidence of moderate-to-severe OA, and patients with unstable knees.

**Table 2:** List of inclusion and exclusion criteria for patients by study

	Paper	Inclusion and exclusion criteria
Actifit	Leroy et al. [14]	*Inclusion* patients between 18 and 50 years of age with stable or stabilised knee with post-meniscectomy pain. Prior meniscectomy had to be partial with an intact meniscal rim and anterior and posterior horns on pre-operative MRI *Exclusion* patients with an alignment defect on standing long-leg radiographs of more than 5° or ICRS 3 and 4 chondral lesions in mirror or extended more than 2 cm^2^
	Dhollander et al. [15]	*Inclusion* an irreparable medial or lateral meniscal tear or partial meniscus loss with an intact rim, skeletally mature male or female patients, from 16 to 50 years of age, a stable knee joint or knee joint stabilisation procedure within 12 weeks of the index procedure, International Cartilage Repair Society (ICRS) classification < 3, no more than three surgeries on the involved meniscus *Exclusion* total meniscus loss or an unstable segmental rim defect, a meniscal root tear, multiple areas of unilateral partial meniscus loss that could not be treated by a single scaffold, ICRS classification > 3, BMI > 35 kg/m^2^ and untreated tibiofemoral malalignment
	Toanen et al. [16]	*Inclusion* an irreparable medial or lateral meniscal tear or partial meniscus loss with an intact rim, skeletally mature male or female patients, from 16 to 50 years of age, a stable knee joint or knee joint stabilisation procedure within 12 weeks of the index procedure, International Cartilage Repair Society (ICRS) classification < 3, no more than three surgeries on the involved meniscus *Exclusion* total meniscus loss or an unstable segmental rim defect, a meniscal root tear, multiple areas of unilateral partial meniscus loss that could not be treated by a single scaffold, ICRS classification > 3, BMI > 35 kg/m*2* and untreated tibiofemoral malalignment
	Monllau et al. [17]	*Inclusion* persistent medial or lateral joint line due to a previous partial meniscus resection, intact outer rim of the meniscus *Exclusion* complete loss of the corresponding meniscus, symptomatic grade III or IV chondral injury in whatever knee compartment, untreated instability, untreated varus or valgus malalignment greater than 5°, inflammatory arthritis, polyurethane allergies, autoimmune disease and pregnancy were excluded
	Filardo et al. [18]	*Inclusion* skeletally mature patients, affected by meniscal loss greater than 25%, intact anterior and posterior meniscus attachments, intact rim at the circumference of the missing meniscus *Exclusion* patients with uncorrected knee axis deviation or instability, allergy to polyurethane, systemic administration of corticosteroids/immunosuppressive agents within 30 days before surgery, osteonecrosis of the index knee, history of infectious, neoplastic, metabolic or inflammatory conditions
CMI		
	Zaffagnini et al. [19]	*Inclusion* irreparable acute meniscal tears requiring partial meniscectomy or chronic prior loss of meniscal tissue (traumatic or degenerative) greater than 25%, intact anterior and posterior attachments of the meniscus, intact rim (1 mm or greater) over the entire circumference of the involved meniscus, anterior cruciate ligament (ACL) deficiencies stabilised at the time of the index surgery, participant between 15 and 60 years of age, contralateral healthy knee *Exclusion* concomitant posterior cruciate ligament (PCL) insufficiency of the involved knee, diagnosis of Outerbridge grade IV, uncorrected malformations or axial malalignment, documented allergy to collagen or chondroitin sulfate of animal origin, systemic or local infection, history of anaphylactoid reaction, systemic administration of any type of corticosteroid or immunosuppressive agents within 30 days of surgery, evidence of osteonecrosis in the involved knee, history of rheumatoid arthritis, inflammatory arthritis, or autoimmune diseases, neurological abnormalities or conditions that would preclude the patient’s requirements for the rehabilitation program, pregnancy
	Steadman et al. [20]	*Inclusion* acute or chronic injuries resulting in loss of at least one-third of the native meniscus but who had an intact meniscus rim of at least 1 mm or greater, stable or stabilised knee at the time of surgery *Exclusion* total meniscus loss, grade IV (full thickness) chondral defects, varus axial malalignment, inflammatory or systemic disease, had known collagen allergies, were diagnosed with autoimmune disease or were pregnant
	Zaffagnini et al. [21]	*Inclusion* irreparable medial meniscus tear at arthroscopy or a previous major loss of meniscus cartilage after partial meniscectomy. Knees had to be stable or surgically stabilised at the time of the implantation procedure. Both traumatic and degenerative loss of meniscus cartilage were included
	Bulgheroni et al. [22]	*Inclusion* combined ACL and CMI implantation from 2001 to 2005 in two hospitals and all patients included in this study had acute or chronic complete ACL rupture associated with irreparable medial meniscus injury requiring partial meniscectomy or with partial defect from previous partial meniscectomy
	Bulgheroni et al. [23]	*Inclusion* irreparable medial meniscus tears with meniscus removal greater than 25% of total meniscus or presence of persistent pain after meniscectomy, according to the instructions for use of the CMI provided by the producer *Exclusion* patients with Outerbridge grade IV chondral lesions, autoimmune diseases, infection, other systemic diseases, collagen of animal origin allergies and aged over 60 years
	Monllau et al. [24]	*Inclusion* persistent medial compartmental joint line pain associated with sizeable meniscus resection or irreparable meniscus tear at arthroscopy. Anterior and posterior meniscus remnants and intact outer meniscal rims were necessary conditions for the procedure. Anterior cruciate ligament (ACL) deficiency was not considered a contraindication if the ligament was reconstructed at the same time as the CMI implantation *Exclusion* Ahlbäck grade > II on the radiographs of the medial tibiofemoral compartment, complete loss of the medial meniscus, lateral meniscus injuries, untreated instability, grade IV chondral lesions, axial deviation greater than 5°, inflammatory arthritis, collagen allergies, autoimmune disease and pregnancy
NUsurface	McKeon et al. [11]	*Inclusion* Previous medial meniscectomy confirmed by MRI and history at least 6 months before the start of study treatment, pain score ≤ 75 on the Knee Injury and Osteoarthritis Outcome Score, ≥ 2 mm intact meniscal rim, age between 30 and 75 years, neutral alignment ±5° of the mechanical axis *Exclusion* Outerbridge grade IV, varus/valgus knee deformity > 5°, knee laxity, level > II ICRS, secondary to previous injury any knee ligament, patellar compartment pain and/or patellar articular cartilage damage, ACL reconstruction performed < 9 months before implantation and body mass index > 32.5

### Clinical outcomes

Clinical outcomes improved in the Actifit, CMI and NUsurface scaffolds (Table 3). The visual analogue score (VAS) and Lysholm, Tegner and KOOS scores improved post-operatively with the Actifit implant. One study did not show a significant improvement in Tegner scores [17], and another did not show a significant improvement in KOOS scores [14]. The NUsurface preliminary data recorded the KOOS score only, which was found to improve across all five domains and were statistically significant, although mean differences were reported instead of absolute values [11]. The CMI scaffold data showed an improvement in the VAS score, Lysholm score and Tegner Score post-operatively. However, one case series of eight patients did not report mean clinical improvement values [21], and another study did not report absolute values [23].

**Table 3:** Outcome score per publication

	Pre-operative VAS score	Post-operative VAS score	Lysholm pre-operative	Lysholm post-operative	Tegner pre-operative	Tegner post-operative	Pre-operative KOOS symptom	Post-operative KOOS symptom	Pre-operative KOOS pain score	Post-operative KOOS pain score	Pre-operative KOOS ADL	Post-operative KOOS ADL	Pre-operative KOOS Sport/REC	Post-operative KOOS Sport/Rec	Pre-operative KOOS QOL	Post-operative KOOS QOL
Actifit																
Leroy [14]	55 ± 20	29 ± 26*	–	–	–	–	69.4 ± 13	68.3 ± 23	62.9 ± 15	76.1 ± 25	72 ± 20	81.7 ± 23	51.2 ± 14	53.5 ± 33	40.9 ± 18	59.9 ± 31*
Dhollander [15]	56.2 ± 21.6	19.3 ± 26.9*	–	–	–	–	52.4 ± 19.7	69.4 ± 20.9*	48.3 ± 20.3	77.2 ± 24.5*	54.4 ± 21.5	80.2 ± 26.1*	19.1 ± 20.0	49.7 ± 34.8*	32.2 ± 14.2	56.9 ± 24.0*
Toanen [16]	54.0 ± 20.7	15.2 ± 19.2*	60.5 ± 19.6	84.5 ± 20.1*	–	–	56.0 ± 19.7	78.2 ± 19.5*	54.2 ± 22.0	78.4 ± 21.3*	61.2 ± 24.0	82.1 ± 21.0*	28.5 ± 24.0	53.9 ± 31.4*	30.9 ± 16.7	56.2 ± 26.4
Monllau [17]	–	–	40.7	78.1*	5.1	5.7	–	–	–	–	–	–	–	–	–	–
Filardo [18]	–	–	–	–	2#	3.5#*	–	–	–	–	–	–	–	–	–	–
Mean	**55.0**	**21.2**	**50.6**	**81.3**	**3.6**	**4.6**	–	–	–	–	–	–	–	–	–	–
CMI																
Zaffagnini [19]	60	﻿12*	–	–	1	4*	–	–	–	–	–	–	–	–	–	–
Steadman [20]	23	11	75	88*	3.4	6*	–	–	–	–	–	–	–	–	–	–
Zaffagnini [21]	–	–	–	–	–	–	–	–	–	–	–	–	–	–	–	–
Bulgheroni [22]	53.5 ± 30.8	14.7 ± 18.7*	57.3 ± 16.9	94.1 ± 8.2*	3	6*	–	–	–	–	–	–	–	–	–	–
Bulgheroni [23]	NR	NR	58	$*	2	$*	–	–	–	–	–	–	–	–	–	–
Monllau [24]	55	20*	59.9	87.5*	–	–	–	–	–	–	–	–	–	–	–	–
Mean	**47.9**	**14.4**	**62.6**	**89.9**	**2.4**	**5.3**	–	–	–	–	–	–	–	–	–	–
NUsurface																
McKeon [9]	–	–	–	–	–	–	NR	Improved by 16.1*$	NR	Improved by 30.4*$	NR	Improved by 26.5*$	NR	Improved by 34.6* $	NR	> 20 points from pre-op*$

Bold represents mean and total values for the individual subgroups of mean data sets

* Statistically significant difference between pre- and post-op value (p<0.05)

# Median instead of mean recorded

$ Absolute value not recorded

## Concurrent procedures performed

All of the Actifit [14–18] and five out of six CMI studies [19, 21–24] had some form of concurrent procedure performed on the operated knees (Table 4). The Actifit studies had 118 (45%) patients undergo a concurrent procedure, and the CMI patients had 53 (45%). The commonest procedure performed for the Actifit patients was a high tibial osteotomy (HTO) (54), followed by an ACL repair (47). Out of 53 patients who had concurrent procedures in the CMI studies, the most common procedure was ACL reconstruction (45), followed by HTO (2). The concurrent procedures for the NUsurface implant have not been recorded, however, the inclusion criteria for the study included patients who have normal leg alignment and had not undergone ACL reconstructions in the prior 9 months [11].

**Table 4:** Concurrent procedures performed with meniscal scaffold implantation

	Paper	Patients with concurrent procedures (% of total)	ACL reconstructions	ACL with microfracture	ACL with HTO	High tibial osteotomy	HTO and microfracture	Microfracture	Other**
Actifit	Leroy [19]	6 (40%)	5	–	–	–	–	–	1
	Dhollander [20]	8 (18%)	4	–	–	4	–	–	–
	Toanen [16]	68 (44%)	29	–	–	43	–	6	–
	Monllau [22]	25(78%)	6	2	1	3	9	3	1
	Filardo [23]	11(69%) *	3	–	–	4	–	3	7
CMI	Zaffagnini [19]	4 (24%)	2	–	–	–	–	2	–
	Steadman [20]	0 (0%)	–	–	–	–	–	–	–
	Zaffagnini [21]	3 (38%)	2	–	–	–	–	1	–
	Bulgheroni [22]	17 (100%)	17	–	–	–	–	–	–
	Bulgheroni [23]	14 (41%)	11	–	–	2	–	1	1
	Monllau [24]	15 (60%)	13	–	–	–	–	1	1

*Multiple procedures performed on patients

**Other: mosaicplasty, PCL repair, lateral releases, loose body removal, chondroabrasion, shaving

Only one Actifit study stratified patients into different sub-groups for patients who had undergone scaffold implantation between those that had undergone ACL reconstructions or HTO [16]. However, it is unclear whether or not there was a cohort of patients who had scaffolds only [16]. They found that at 5 years, patients who had undergone an ACL reconstruction had statistically significant improvements in VAS, Lysholm and KOOS symptoms scores [16]. Comparison of the HTO versus patients without HTO showed no statistically significant differences in clinical outcome scores at 5 years [16].

### Failures

The failure rates for Actifit, CMI and NUsurface scaffolds are summarised in the figure above. Failure rates for the Actifit ranged from 6.3% to 31.8% with a total failure rate of 18.0% at 66.8 months; for the CMI, the range was 0–11.8% with a total failure rate of 6.5% at 97.1 months. The NUsurface failure rate was 16.9% at 12 months from one study (Table 5).

**Table 5:** Failures

	Paper	Failures/patients	M/L	
Actifit	Leroy [19]	3/15 (16.7%)	3/0	Removal of implant
	Dhollander [20]	14/44 (31.8%)	8/6	Three removals of the scaffold, five conversions to a meniscal transplant, four converted to UKR, two converted to TKR
	Toanen [16]	23/137 (16.8%)	NR	Ten scaffolds broke, seven were converted to meniscal allografts, four converted to UKR, two converted to TKR
	Monllau [22]	3/32 (9.4%)	NR	Removal of scaffold
	Filardo [23]	1/16 (6.3%)	NR	Need for re-operation due to symptoms related to index defect
Total		44/244 (18.0%)		
CMI	Zaffagnini [19]	2/17 (11.8%)	2/0	Re-operation
	Steadman [20]	0/8 (0%)	n/a	Complications related to implant
	Zaffagnini [21]	0/8 (0%)	n/a	Not recorded
	Bulgheroni [22]	NR	n/a	Not recorded
	Bulgheroni [23]	2/34 (5.9%)	2/0	Implant failure, or dissolved
	Monllau [24]	2/25 (8%)	2/0	Infection or mechanical failure of implant
Total		6/92 (6.5%)		
NUsurface	McKeon [9]	11/65(16.9%)	11/0	Further surgery: three device repositioning, three replacement of device, two removals, one UKA, two unplanned arthroscopies

## Discussion

The current information from literature on meniscal scaffolds is insufficient to suggest that they could potentially improve clinical outcomes in patients post-meniscal resection. This is largely due to the high proportion of concurrent procedures performed at surgery in both the Actifit and CMI studies. Only one Actifit [16] and one CMI study [20] stratified concurrent procedures into sub-groups for separate statistical analysis. This makes meaningful direct comparison of clinical outcomes following isolated meniscal defects treated by meniscal scaffolds not possible.

The failure rates for Actifit and NUsurface were higher than that for the CMI implant. The mean failure rate for Actifit was 18.0% (range of 6.3–31.8%), subsequent procedures included removal of the implant, conversion to MAT, scaffold breakage, and conversion to UKR/TKR. The Actifit implant was used for both medial and lateral meniscal substitution, whereas the CMI and NUsurface scaffolds were used only for the medial side. The failure rate for CMI was 6.5% (range 0–11.8%), with subsequent recorded procedures not consistently recorded. Lastly, the failure rate for NUsurface was 16.9% after 12 months, with patients undergoing device repositioning, replacement, removals and conversion to UKR [11]. Survivorship of the Actifit and CMI scaffolds is comparable to medial MAT, which have been shown to be 86.2% at 5 years [25], and 73.5% at 10 [26]. Given the variability in recording of failures and volume of concurrent procedures, failures were not sub-analysed for individual concurrent procedures. This issue has been recognised previously in the literature [17], and further investigation into outcomes and complications associated with concurrent procedures would be beneficial.

### Secondary prevention of osteoarthritis

The evidence for the chondroprotective effects, and thus secondary prevention of OA of meniscal scaffolds, is not sufficient. One study showed that at 5 years, the Actifit scaffold showed a small increase in the volume of meniscal tissue [22]. An alternate study evaluated International Cartilage Repair Society (ICRS) scores and demonstrated a worsening of the cartilage status of the Actifit implant in 7/15 patients who underwent post-operative MRI scans [15].

For CMI, one study which performed MRI scans to assess the Yulish scores (an MRI scoring system for cartilage defects) showed a normal cartilage signal in over 60% of patients at 5 years post-operation [23]. Second-look arthroscopies showed that the implant was present but reduced in size [23]. Another study assessed progression of osteoarthritis in CMI patients using Rosenberg X-ray views [24]. They found that only one of their patients progressed from an Ahlbäck grade 0 to Ahlbäck grade 2 [24]. However, there are studies that have shown no statistically significant improvement in Yulish scores post CMI scaffold implantation [19]. Overall, there is limited evidence of the chondroprotective effects of the Actifit and CMI meniscal scaffolds.

There are several limitations of this review. Firstly, there was a high volume of concurrent procedures performed at the time of meniscal implantation. In addition, there was also a significant variability in the clinical outcomes reported. These factors make direct meaningful comparison of clinical outcomes impossible. In addition to this, the inclusion criteria for the individual studies included in this review also varied. Given the high level of heterogeneity, meta-analyses and statistical comparison were felt not to be appropriate at this stage. To evaluate clinical outcomes and failure rates, further randomised control trials with comparable clinical outcomes and without concurrent procedures are required. This review is also susceptible to publication bias, as there is significant variability in the criteria for failure in each of the studies.

## Conclusion

On the basis of current evidence, the use of meniscal scaffolds as a sole treatment for partial meniscal defects cannot be recommended, owing to the relatively high failure rate and paucity of clinical data. The evidence for their chondroprotective effects, and thus prevention of secondary OA, remains inconclusive. Further high-quality comparative randomised control trials are required before meniscal scaffolds can be recommended for routine clinical use.

## Data Availability

Data sharing not applicable to this article as no datasets were generated or analysed during the current study.
